# Preclinical Characterization of Antioxidant Quinolyl Nitrone QN23 as a New Candidate for the Treatment of Ischemic Stroke

**DOI:** 10.3390/antiox11061186

**Published:** 2022-06-16

**Authors:** Emma Martínez-Alonso, Alejandro Escobar-Peso, Alicia Aliena-Valero, Germán Torregrosa, Mourad Chioua, Rocío Fernández-Serra, Daniel González-Nieto, Youness Ouahid, Juan B. Salom, Jaime Masjuan, José Marco-Contelles, Alberto Alcázar

**Affiliations:** 1 Department of Research, Hospital Universitario Ramón y Cajal, IRYCIS, 28034 Madrid, Spain; emma.martinez@hrc.es (E.M.-A.); alejandro.escobar@hrc.es (A.E.-P.); 2 Unidad Mixta de Investigación Cerebrovascular, Instituto de Investigación Sanitaria La Fe, Universidad de Valencia, 46026 Valencia, Spain; alicia_aliena@iislafe.es (A.A.-V.); torregrosa_ger@gva.es (G.T.); salom_jba@gva.es (J.B.S.); 3 Laboratory of Medicinal Chemistry (IQOG, CSIC), 28006 Madrid, Spain; m.chioua@csic.es (M.C.); jlmarco@iqog.csic.es (J.M.-C.); 4 Center for Biomedical Technology, Universidad Politécnica de Madrid, 28223 Pozuelo de Alarcón, Spain; rocio.fernandez@ctb.upm.es (R.F.-S.); daniel.gonzalez@ctb.upm.es (D.G.-N.); 5 Centro de Investigación Biomédica en Red of Bioengineering Biomaterials and Nanomedicine (CIBER-BBN), 28029 Madrid, Spain; 6 Isquaemia Biotech SL, 28102 Alcobendas, Spain; youness@isquaemiabiotech.com; 7 Department of Physiology, Universidad de Valencia, 46010 Valencia, Spain; 8 Department of Neurology, Hospital Universitario Ramón y Cajal, IRYCIS, 28034 Madrid, Spain; jaime.masjuan@salud.madrid.org; 9 Department of Medicine, Facultad de Medicina, Universidad de Alcalá, 28871 Alcalá de Henares, Spain

**Keywords:** antioxidants, brain ischemia, ischemic stroke, neuroprotection, quinolyl nitrones, pharmacokinetics, reactive oxygen species

## Abstract

Nitrones are encouraging drug candidates for the treatment of oxidative stress-driven diseases such as acute ischemic stroke (AIS). In a previous study, we found a promising quinolylnitrone, QN23, which exerted a neuroprotective effect in neuronal cell cultures subjected to oxygen–glucose deprivation and in experimental models of cerebral ischemia. In this paper, we update the biological and pharmacological characterization of QN23. We describe the suitability of intravenous administration of QN23 to induce neuroprotection in transitory four-vessel occlusion (4VO) and middle cerebral artery occlusion (tMCAO) experimental models of brain ischemia by assessing neuronal death, apoptosis induction, and infarct area, as well as neurofunctional outcomes. QN23 significantly decreased the neuronal death and apoptosis induced by the ischemic episode in a dose-dependent manner and showed a therapeutic effect when administered up to 3 h after post-ischemic reperfusion onset, effects that remained 11 weeks after the ischemic episode. In addition, QN23 significantly reduced infarct volume, thus recovering the motor function in a tMCAO model. Remarkably, we assessed the antioxidant activity of QN23 in vivo using dihydroethidium as a molecular probe for radical species. Finally, we describe QN23 pharmacokinetic parameters. All these results pointing to QN23 as an interesting and promising preclinical candidate for the treatment of AIS.

## 1. Introduction

According to the WHO’s Global Health Estimates Report of 2019, stroke represents the second largest cause of death worldwide [1], with the ischemic subtype accounting for approximately 80–85% of registered cases [1]. Brain ischemia is usually caused by a thrombus occluding a blood vessel, or by a systemic hypoperfusion, and is characterized by a decrease in the blood supply to the brain, which triggers a set of processes known as ischemic cascade within the cerebral tissue [2]. Depending on the severity of the ischemic insult (i.e., level and duration of blood reduction), and the particular area of the brain affected, an ischemic insult may lead to the loss of cell function and, eventually, cell death [2]. Thus, the first therapeutic approach must be aimed at recovering normal blood flow levels to the tissue affected. Currently, mechanical thrombectomy or pharmacological thrombolysis (e.g., with alteplase or tenecteplase) may afford recanalization in eligible patients [3]. However, despite being essential to stop further cell death, tissue reperfusion does not ensure a total recovery of cell function. In fact, reperfusion may also exacerbate the damage produced within the ischemic penumbra in what is called “ischemia-reperfusion damage” [4].

One of the main actors playing a role in ischemic tissue recovery following reperfusion is oxidative stress. As a consequence of the massive influx of oxygen upon the previously compromised tissue of the penumbra, radical species production overwhelms the endogenous antioxidant systems and exerts structural and functional damage, hindering tissue recovery and inducing cell death [5,6]. Therefore, antioxidant therapies able to reduce the impact of this damage have been highly sought after. The nitrone functional group (i.e., *N*-oxide of an imine) has been a widely studied chemical group in the search for drugs aimed at reducing oxidative stress. Long described as radical traps due to their radical species scavenging activity [7], nitrones were proposed early on as potentially therapeutic antioxidant compounds [8,9]. In particular for ischemic stroke treatment, the widely known nitrone NXY-059 (Cerovive, developed by AstraZeneca) served for many years as the antioxidant nitrone paradigm. As a more soluble and potent derivative of the original *N*-*tert*-Butyl-α-phenylnitrone (PBN), NXY-059 was reported to reduce lesion volume and motor impairment in preclinical phases, including several animal species and experimental stroke models [10]. Phase I and II clinical trials reported a good pharmacokinetic profile [11,12,13], but a lower brain permeability than original PBN was reported, even though it was increased as a consequence of the ischemic episode [14]. These results, along with no remarkable safety concerns, promoted NXY-059 to Phase III clinical trials, SAINT I and SAINT II, whose pooled results eventually revealed that NXY-059 did not afford a significant improvement in stroke patients when compared with placebo [15]. Posterior reviews and meta-analysis pointed out an overestimation of efficacy in preclinical trials, which presented several drawbacks that avoided the translation of the preclinical results of NXY-059 to clinical practice [10], apart from unresolved enquiries regarding NXY-059’s true mechanism of action [16]. Currently, numerous guidelines highlight the need for a rigorous preclinical study including relevant aspects in the clinical practice treatment of particular diseases and their outcomes early in the development of new neuroprotectant drugs [17].

**Scheme 1:**
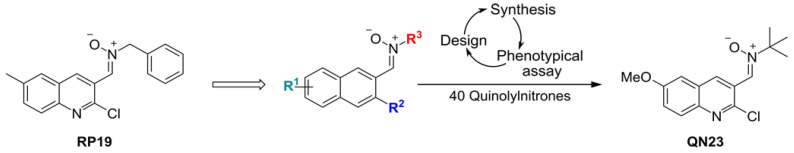
Development of QN23.

In spite of it, NXY-059 served to make clear the therapeutic potential of nitrones as candidates for the treatment of different diseases, especially ischemic stroke. For many years, our group has been developing new nitrone-derived compounds based on diverse scaffolds as potential drug candidates for the treatment of the ischemic neuropathology. Apart from the cholesterol-derived nitrone candidate ISQ-201 [18], our group identified a set of nitrones bearing a quinoline core as interesting neuroprotective compounds in oxygen and glucose deprivation (OGD) injury on primary neuronal cultures [19]. One of these quinolyl nitrones, named RP19, was identified as the most potent of the set, with a promising neuroprotective effect against OGD insult, and thus prompted its study on in vivo stroke experimental models, confirming its neuroprotective effect [20]. Further exploration of the quinoline core and screening of more than 40 quinolyl-derived compounds in our phenotypical OGD model has led to new interesting candidates, with the one identified as QN23 being our current lead quinolyl nitrone [21,22]. QN23 presented higher protection and tolerability than previous RP19, and higher potency than NXY-059, on primary neuronal cultures subjected to OGD. Furthermore, QN23 had a remarkable antioxidant profile, being particularly strong against hydroxyl radical, which was complemented by studies in primary neuronal cultures in which QN23 reduced reactive oxygen species (ROS) and lipid peroxidation levels [22]. The in vivo study of its neuroprotective activity revealed decreased levels of neuronal death and apoptosis and an improvement in neurological function, after intraperitoneal injection of QN23 at the onset of post-ischemic reperfusion [22]. In addition, the study of the anti-inflammatory activity of QN23 in the carrageenan-induced rat paw edema experimental model showed anti-inflammatory properties [22]. In our search for new therapeutic compounds for the treatment of ischemic stroke, Scheme 1 shows QN23 development as the lead compound among the new quinolyl drug candidates. In order to keep with its development, new evidence of antioxidant activity and relevant preclinical characterization studies of QN23 are described in this report.

In particular, in the present study we assess the effectivity of QN23 when administered intravenously, in contrast to previous studies based on intraperitoneal administration. We also report the dose–response study of QN23 in order to establish its tolerability and efficacy in vivo, and we evaluate the infarct size and neurofunctional impairment reductions by QN23 treatment in an alternative transient middle cerebral artery occlusion (tMCAO) experimental stroke model performed by an independent laboratory. Another aspect of importance in the development of new therapeutic candidates is the assessment of a clinically useful therapeutic window [3]. In this report, a therapeutic window study of QN23 was also included. Finally, and considering the dramatic increase in stroke incidence in younger individuals (i.e., younger than 55 years) being reported recently [23], an assessment of the effect of any potential treatment on functional sequelae later in time after the ischemic episode is required. These adults may eventually suffer from compromising neurofunctional or neuropsychiatric outcomes, long after the ischemic episode but still early in life [24]. Due to this, the assessment of a long-term effect should also be considered early in preclinical phases of drug development [17]. Therefore, long-term efficacy of QN23 was assessed 3 months after the ischemic insult.

## 2. Materials and Methods

### 2.1. Compounds

Quinolyl nitrone QN23 was synthesized as described [22]. NXY-059 was supplied by TargetMol (Boston, MA, USA) (T6201).

### 2.2. Animal Model of Global Cerebral Ischemia, Experimental Design, and Treatment

Transient forebrain ischemia was induced in adult male Wistar rats (10–12 weeks, Charles River, L’Arbresle, France) by the standard four-vessel occlusion (4VO) model previously described [25,26]. Briefly, animals were anesthetized by intraperitoneal injection of a combination of ketamine, diazepam, and atropine (62.5, 5.0, and 0.25 mg/kg, respectively); positioned in a stereotactic device; and both vertebral arteries were cauterized. The next day, anesthesia was induced with 4% isoflurane and maintained at 2–2.5% isoflurane for carotid artery dissection. After anesthesia withdrawal, the carotid arteries were occluded with an atraumatic vascular clamp to induce brain ischemia. During the entire surgical procedure, temperature was monitored at 2 min intervals and kept at 37 °C. After 15 min of ischemia, the carotid arteries were unclamped for brain reperfusion. The animals were studied after 5 days of reperfusion (R5d), or after 11 weeks of reperfusion (long-term studies), and then they were euthanized under deep anesthesia. Animals from the sham control group were handled in the same way, but without occlusion of the carotid arteries. Ischemic animals were treated with vehicle solution (ethanol/polyethylene glycol 400/saline, 1:300:200 *v/v*), or with nitrones QN23 or NXY-059 in vehicle solution, as a single injection through the tail vein at the onset of reperfusion. For the therapeutic window experiments, the animals were also treated at reperfusion times 1, 3, and 6 h after ischemia. The treatments were prepared by an independent researcher and administered with concealed allocation. Treatments were administered in a randomized order obtained by a computer-based randomization program. To determine the sample size, a power analysis (http://www.biomath.info/power/ttest.htm, accessed on 12 March 2020) with significance level set at 0.05 and statistical power set at 0.8 (80%) rendered a sample size of <6 subjects per group. A total of 120 ischemic animals (30 animals treated with vehicle, 60 treated with QN23, and 30 treated with NXY-059) and 9 sham control animals were included in these studies. Additionally, 13 animals were excluded from the study: 6 animals were not ischemic and 7 animals died within the 24 h after the surgical procedure (5 vehicles, 1 QN23-treated at 1.0 mg/kg, and 1 QN23-treated at 2.0 mg/kg). The Animal Care Ethics Committee of the Hospital Universitario Ramón y Cajal (Madrid, Spain) authorized all the procedures involving the animal experimentation reported here (authorization number 04/2020). These experiments were performed according to ARRIVE guidelines and the legislation on the protection of animals used for scientific purposes in Spain (RD 53/2013) and the EU (Directive 2010/63/EU). The study was designed and conducted according to the STAIR guidelines [3,17] regarding physiological monitoring, sample randomization, predefined exclusion criteria, allocation concealment, blinded assessment of outcomes, dose–response definition, and a conflict-of-interest statement.

### 2.3. Evaluation of Neurological Deficits

After 5 days of post-ischemic reperfusion (R5d), neurological deficits were evaluated in the animals by a researcher blind to the treatment allocation. General status and mobility impairments were quantified according to a scale previously validated in an entire cohort of R5d animals (*n* = 20) [27]. This scale ranges from 0 (best, non-operated control animal) to 10 (animal with a high neurofunctional deficit).

### 2.4. Behavioral Tests

Long-term assessment of neurological damage after the ischemic episode was performed by the evaluation of the spontaneous exploratory activity (spatial recognition) and spatial memory skills in the Y maze [28].

For the assessment of the exploratory activity and spatial recognition, control animals and ischemic animals treated with vehicle, QN23, or NXY-059 were placed in the center of the maze and allowed free exploration for 8 min. An independent researcher blind to the treatment allocation registered the total number of arms that the animal entered indistinctly (arm entries), and the times that the animal explored the three different arms of the maze consecutively (alternations or triads).

For the assessment of spatial memory, experiments consisted of two trials. For the first trial (training), control and ischemic animals treated with vehicle or QN23 were placed into one of the arms (start arm) and allowed to explore the Y maze for 10 min with one of the arms closed. One hour after the training trial, the animals were returned to the Y maze and the second trial was conducted. The animals were placed in the same start arm as in the training trial, but now with the remaining two arms of the maze available for exploration for 3 min. A researcher blind to the treatment allocation registered the first choice of arm for entry (novel or old).

### 2.5. Brain Sections

After 5 days of reperfusion (R5d), or at 11 weeks of reperfusion in long-term studies, animals were euthanized by transcardiac perfusion via the left ventricle under deep anesthesia. First, blood was washed out with 200 mL of ice-cold saline medium, followed by fixation with 4% (*w/v*) paraformaldehyde solution in PBS for brain preservation. Then, the brains were carefully removed from the cranial cavity and placed in 4% (*w/v*) paraformaldehyde solution in PBS overnight at 4 °C. The next day, the brains were sequentially placed in 10%, 20%, and 30% (*w/v*) sucrose in PBS until sunken, and finally embedded in Tissue-Tek O.C.T. (Sakura Finetek, Torrance, CA, USA) before freezing at −80 °C. Brain coronal sections containing the hippocampus region were obtained by cryostat sectioning at the level of interaural line +5.7 ± 0.2 mm on Real Capillary Gap microscope slides (Agilent Dako, Santa Clara, CA, USA).

### 2.6. Neuronal Death Evaluation

Neuronal death was determined by Fluoro-Jade B staining [25] of brain cryosections (10 µm thick) obtained from ischemic animals after 5 days of post-ischemic reperfusion and fixed as described above. The cerebral and lateral cortex fields and the hippocampal CA1 subfield from a given section were analyzed with fluorescence microscopy (40× objective) and dead cells (in green) were counted using a grid of 330 × 220 µm, with a total area of 1.017 mm^2^ per section analyzed and two sections per brain sample. Fluorescence images were digitized with a color CCD camera (1280 × 960 pixel resolution). Data analysis was independently carried out by two researchers blind to the treatment allocation.

### 2.7. TUNEL Assay

Neuronal apoptosis was determined in brain sections from ischemic animals by the Terminal deoxynucleotidyl transferase-mediated dUTP Nick-End Labeling (TUNEL) assay (Promega, Madison, WI, USA), following the supplier protocol with little modifications. Briefly, 5 µm-thick coronal brain sections obtained as described above were post-fixed with 4% (*w/v*) paraformaldehyde solution in PBS (5 min, at room temperature). After washing in PBS (3×, 5 min), sections were permeabilized with 0.1 M sodium citrate (pH 6.0) at 95 °C for 1 min, quickly cooled in ice, and washed in PBS at room temperature. Then, brain sections were blocked (0.1 M Tris-HCl, pH 7.5, 20% fetal bovine serum and 3% bovine serum albumin), washed in PBS, and fixed again with 4% (*w/v*) paraformaldehyde solution in PBS (5 min, at room temperature). After washing in PBS (3×, 5 min), sections were incubated with the kit buffer for 10 min, followed by incubation with terminal deoxynucleotidyl transferase (TdT) and fluorescein-12-dUTP (1.5 h, 37 °C). The reaction was stopped by extensive washing in saline–sodium citrate buffer (30 mM sodium citrate in 0.3 M sodium chloride) at room temperature, followed by washing in PBS (3×, 5 min). Finally, sections were mounted with coverslips in antifade buffered glycerol solution containing 4.6 mM *p*-phenylenediamine and 30 µM bisbenzimide (Hoechst 33342) for nuclear staining.

Green-labeled apoptosis-positive cells were counted as in Fluoro-Jade B staining (see above). Data analysis was carried out by two independent researchers blinded to the treatment allocation.

### 2.8. Immunohistochemistry for Neuronal Viability Evaluation

Brain cryosections from ischemic animals after 11 weeks of reperfusion were used after fixation to detect neuronal viability by immunohistochemistry. Sections (10-µm thick) were subsequently post-fixed with 4% (*w/v*) paraformaldehyde solution in PBS for 5 min at room temperature, washed in PBS (3×, 5 min), and permeabilized with 10 mM sodium citrate (pH 6.0) for 3 min at 95 °C, followed by cooling at room temperature for 20 min and washing in PBS (3×, 5 min). Then, brain sections were blocked (5% heat-inactivated donkey serum, 0.1% Triton X-100 in PBS) for 1 h at room temperature and incubated with mouse monoclonal anti-S6 protein antibody (Cell Signaling Technology, Danvers, MA, USA) overnight at 4 °C. After washing in PBS (3×, 5 min), sections were incubated with X Red-conjugated anti-mouse antibody (Jackson ImmunoResearch, West Grove, PA, USA) for 1 h at room temperature and washed again in PBS. Sections were mounted with coverslips in antifade buffered glycerol solution containing 4.6 mM *p*-phenylenediamine and 30 µM bisbenzimide (Hoechst 33342, Thermo Fisher Scientific, formerly Invitrogen, Waltham, MA, USA) for nuclear staining. The fields of the hippocampal CA1 region of a given section were examined by fluorescence microscopy. Images of more than 6 different fields within a given section were acquired with a 40× objective, and Image-J software (version 1.53, National Institute of Health, Bethesda, Maryland, USA) was used for quantitative analysis of the number of S6-labeled cells per field in the area of interest.

### 2.9. Imaging of Cerebral ROS in Brain Ischemia

In order to evaluate the antioxidant capacity of QN23 in vivo, the fluorescent probe dihydroethidium (DHE) was used for superoxide and hydrogen peroxide detection according to the experimental procedure described in [29], with minor modifications. Animals subjected to global cerebral ischemia (Wistar male rats of 280–300 g) were treated with vehicle or QN23 at the onset of the reperfusion period, and after 2 h of reperfusion DHE (3.0 mg/kg; 0.9 mg/mL in DMSO/polyethylene glycol 400/saline, 2.5:50:47.5 *v/v*) was intravenously injected (1 mL). DHE (Sigma-Aldrich, Merck KGaA, Darmstadt, Germany) was freshly weighted and dissolved just before the injection to avoid undesired air-driven oxidation. At 6 h of reperfusion after ischemia (4 h after DHE injection), the animals were euthanized and perfused with saline and 4% (*w/v*) paraformaldehyde in PBS. The untreated control animals were also injected with DHE and euthanized and perfused in the same way at 4 h after DHE injection.

Rat brains were excised and kept in the same solution in the dark at 4 °C for 2 h, after which paraformaldehyde solution was replaced by 30% sucrose and kept in the dark at 4 °C until brain sinking. Two days after the surgery, the brains were embedded in Tissue-Tek O.C.T. (Sakura Finetek), frozen at −80 °C, and cryo-sectioned at the level of interaural line +5.7 ± 0.2 mm on Real Capillary Gap microscope slides (Dako). Ten µm-thick brain sections were mounted with coverslips in Fluoroshield^®^ with DAPI (Sigma-Aldrich, Merck KGaA, Darmstadt, Germany).

Hippocampal CA1 subfields and cortical fields from a given brain section were randomly selected and analyzed with fluorescence microscopy (20× objective), and the selected images (425 × 340 μm) were digitized with a color CCD camera (1280 × 1024 pixel resolution). Fluorescence intensity (in red) of ROS-reacted DHE was evaluated and normalized to the number of DAPI-stained nuclei by using an image analysis software (Image-J 1.53). At least four images per brain sample, and 3 to 6 brains per experimental group, were evaluated and averaged. Treatment information was kept concealed throughout the study.

### 2.10. Animal Model of Transient Focal Cerebral Ischemia

For the transient focal cerebral ischemia experimental model we used 12-week-old male Wistar rats (300–350 g, Charles River), housed under standard conditions with food and water ad libitum. The animals were anesthetized by an intraperitoneal injection of a mixture of 100 mg/kg ketamine, 5 mg/kg diazepam, and 0.3 mg/kg atropine. Anesthesia was maintained during the surgical procedure by inhalation of 1–1.5% sevoflurane in 80% medicinal air plus 20% O_2_. For the transient occlusion of the right middle cerebral artery (tMCAO, 60 min), we followed the intraluminal suture procedure as originally described [30], adapted to our experimental setup [31]. The procedure included continuous monitoring of cortical perfusion (CP, by Laser-Doppler flowmetry), arterial blood pressure, and core temperature, and discontinuous measurement of glycemia levels during preischemia (basal), ischemia, and reperfusion stages. Buprenorphine (subcutaneous, 0.05 mg/kg) was used as an analgesic. Forty-eight hours after the ischemic insult, the animals were euthanized by intracardiac injection of potassium chloride (200 mg/kg) under deep anesthesia, and the brains were obtained.

A power analysis (see above) provided a sample size of 6 subjects per group. A total of 35 animals was used in this study. The animals were randomly assigned to four experimental tMCAO groups, treated with (1) vehicle; (2) QN23, 1.5 mg/kg; (3) QN23, 2.5 mg/kg; and (4) QN23, 4 mg/kg. QN23 was dissolved in vehicle and injected through the femoral vein 20 min after the end of tMCAO. Nine animals were excluded from the study, based on predefined criteria, because of death within the first 24 h after the procedure (1 vehicle, 1 QN23-treated at 2.5 mg/kg, and 4 QN23-treated at 4.0 mg/kg) or due to no infarct after the procedure (1 vehicle, 1 QN23-treated at 1.5 mg/kg, and 1 QN23-treated at 2.5 mg/kg). Experiments were conducted in compliance with the legislation on the protection of animals used for scientific purposes in Spain (RD 53/2013) and the EU (Directive 2010/63/EU). The protocols were approved by the Animal Experimentation Ethics Committee from IIS La Fe (Valencia, Spain, authorization number 2020/VSC/PEA/0076). The study was designed and conducted according to the STAIR/RIGOR guidelines [3,17] regarding physiological monitoring, simple randomization, predefined exclusion criteria, allocation concealment, blinded assessment of outcomes, dose response definition, and a conflict-of-interest statement.

### 2.11. Evaluation of Neurofunctional Score in Transient Focal Cerebral Ischemia

Twenty-four and 48 hours after the ischemic insult, the rats’ neurofunctional status was evaluated following the procedure previously reported [32]. The severity of functional deficits was determined with the sum of the score of four tests assessing (a) spontaneous activity (moving/exploring = 0, no moving or moving only when pushed = 1), (b) circling to the left (none = 0, circling when elevated by the tail and pushed = 1, circling without displacement, spinning = 2), (c) parachute reflex: protective abduction of forelimbs (symmetrical = 0, asymmetrical, contralateral forelimb retracted = 1), and (d) resistance to left forepaw stretching (not allowed = 0, allowed = 1). The total score ranged from 0 (no neurological deficits) to 5 (highest neurological deficits) or 6 (death). Neurofunctional evaluation was performed by an independent researcher blind to the treatment allocation.

### 2.12. Evaluation of Infarct Volume in Transient Focal Cerebral Ischemia

Brain infarct volume was determined by the 2,3,5-triphenyltetrazolium chloride (TTC; Sigma-Aldrich, Merck KGaA, Darmstadt, Germany) vital staining method [33], followed by morphometric analysis [31] by a researcher blind to the treatment allocation. Briefly, rats were euthanized and the brain was sliced in seven 2 mm-thick coronal sections, which were immersed in a 2% solution of TTC in saline solution at 37 °C for 10 min and fixed in 10% phosphate-buffered formalin (pH 7.4) overnight. Digital photographs were taken (Olympus CAMEDIA C-5050, Tokyo, Japan) and the border between infarcted and non-infarcted tissue was outlined with an image analysis system (Image-J software). In order to correct for the influence of edema, the infarcted area was calculated as follows: corrected infarct area = infarct area × (contralateral hemisphere area / ipsilateral hemisphere area). The corrected infarct area was measured on both the anterior and the posterior sides of each slice and averaged; the corrected infarct volume was calculated by multiplying the average corrected infarct area by the thickness of the slice (2 mm), and the total corrected infarct volume by adding up the corrected infarct volume of the seven slices. The operational sequence was applied separately to the cortex and striatum as well as to the sum of both. The subtraction between the raw and corrected infarcted hemisphere equaled the edema volume.

### 2.13. Pharmacokinetic and Toxicity Studies

For the toxicokinetics study of QN23, female Sprague Dawley rats (9–10 weeks, Envigo, Barcelona, Spain) were divided into three groups (*n* = 6 per group) and treated intravenously with different concentrations of QN23 (2.0, 6.0, and 18 mg/kg), in vehicle solution (ethanol/polyethylene glycol 400/saline, 1:200:60 *v/v*), as a single bolus of 1 mL injection at 3 mL/min. Samples of plasma were taken at 0.25, 0.5, 1, 2, 4, and 8 h after the first administration (on day 1) and QN23 concentration was determined by liquid chromatography–mass spectrometry in tandem (LC-MS/MS) analysis for pharmacokinetic study. For the determination of the toxicokinetic parameters, a noncompartmental analysis was performed using the Phoenix 64 WinNonlin software (version 8.0, Certara, Sant Louis, MO, USA). This study was carried out by Swiss BioQuant AG (Reinach, Switzerland). For the toxicity study of QN23, rats were divided into four groups (*n* = 5 per group) and treated intravenously with vehicle (control group) or different concentrations of QN23 (2.0, 6.0, and 18 mg/kg) as a single bolus injection in a 4-day repeated dose as described above. Pathology analyses, comprising hematology, clinical biochemistry, and coagulation, were performed, and heart, kidney, liver, and spleen necropsies were collected for macroscopic analysis. This study was carried out by Vivotecnia Research S.L (Madrid, Spain).

### 2.14. Statistical Analysis

Data from each animal and experimental condition were independently analyzed, and their averaged values were used for statistical analysis. Data are represented as mean ± SE. For comparison between multiple concentrations or experimental groups, an analysis of variance (ANOVA) or non-parametric Kruskal–Wallis test (for neurological scoring variables) was performed, followed by a post-hoc test (Dunnett’s or Dunn’s test, respectively) to compare with control group means by pairs when ANOVA or Kruskal–Wallis tests were significant. The statistical significance level was set at α = 0.05, and the Prism statistical package (version 5.0, GraphPad Software, San Diego, CA, USA) was used.

## 3. Results

### 3.1. Dose–Response Study in Transient Cerebral Ischemia

Previous assessment of the effective dose of QN23 for neuroprotection in primary neuronal cultures subjected to OGD reported an optimal range of concentration of 0.3–250 µM, with 100 µM being the most effective, when applied at the onset of the recovery period after OGD [22]. In addition, previous studies of the effect of QN23 in vivo showed significant neuroprotective effect, as observed by neuronal death and neuronal apoptosis assays, when administered at 1.5 mg/kg intraperitoneally [22].

**Figure 1:**
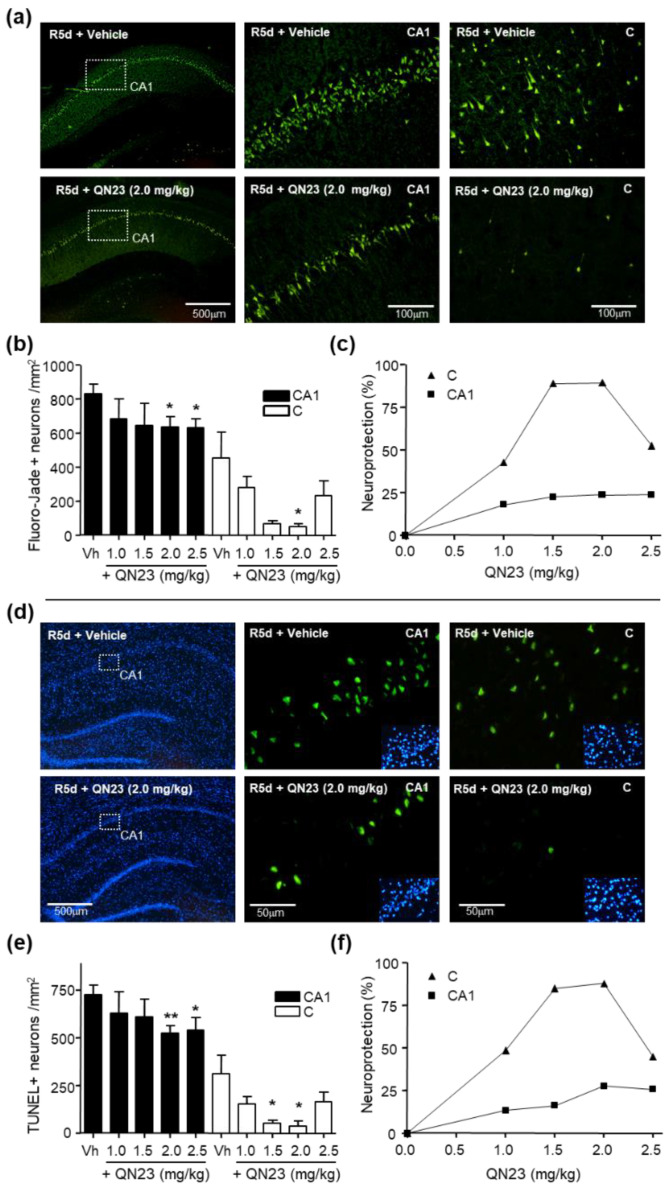
Dose–response curve of QN23. Ischemic animals were treated with vehicle or QN23 (1.0–2.5 mg/kg) by intravenous injection at reperfusion onset after global cerebral ischemia, and the effect of QN23 was studied by assessing neuronal death and apoptosis after 5 days of reperfusion (R5d). (**a**) Representative images of Fluoro-Jade B-stained brain sections from vehicle- or QN23-treated animals (R5d + vehicle and R5d + QN23, respectively). Fluoro-Jade B-positive dead neurons were visualized by fluorescence microscopy (in green) in the hippocampal CA1 (CA1) and cortical (C) regions, and counted as described in the Materials and Methods section. (**b**) Bar graph representation of the neuronal death quantified in brain sections stained with Fluoro-Jade B in CA1 and C regions. Data represent the mean of 9 independent animals treated with vehicle, and 6–7 independent animals per QN23 treatment group; error bars indicate the SE. * *p* < 0.05 and ** *p* < 0.01, compared with the R5d + vehicle (Vh) by Dunnett’s post-test after ANOVA. In (**c**), data from (**b**) are represented as neuroprotective activity of QN23. Neuroprotection was defined as the effect achieved relative to the neuronal death value observed in the R5d + vehicle group (Vh) (0% value) and to the absence of neuronal death (100% value). Data are represented as mean ± SE. (**d**) Brain sections as in (**a**) were used for apoptosis detection by TUNEL assay and visualized by fluorescence microscopy (in green) in CA1 and C regions. Images are representative of the TUNEL assay, with the corresponding Hoechst counterstaining. TUNEL-positive cells were counted in CA1 and C regions as described in the Materials and Methods section. (**e**) Bar graph representation of neuronal apoptosis quantification, performed as in (**b**). In (**f**), data from (**e**) are represented as in (**c**).

**Figure 2:**
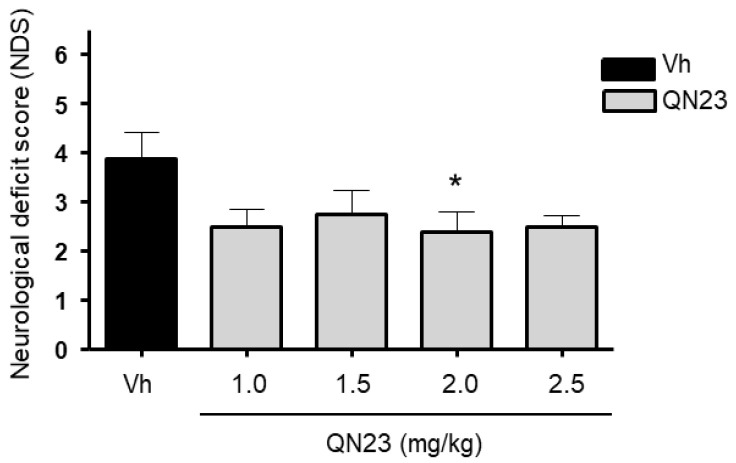
Neurodeficit score outcomes after cerebral ischemia in the dose–response study of QN23. Bar graph representation of the neurological deficit score (NDS) in ischemic animals at 5 days of reperfusion after ischemia. Animals were treated with vehicle (Vh) or QN23 in 1.0, 1.5, 2.0, or 2.5 mg/kg by an intravenous injection at reperfusion onset after cerebral ischemia. A total of 6–9 independent animals (as described in Figure 1) were averaged per group; error bars indicate the SE, * *p* < 0.05, compared with vehicle by Dunn’s post-test after the non-parametric Kruskal–Wallis test.

In the following section, we describe the effect of different doses of QN23 (1.0, 1.5, 2.0, and 2.5 mg/kg) administered intravenously (see Materials and Methods, Section 2.2). In order to explore this wider range of concentrations, solubility studies were carried out before the in vivo experiments in order to assess a proper pharmacological formulation. For the highest concentrations, QN23 was not soluble in our usual ethanolic vehicle (ethanol/saline, 1:9 *v/v*). After exploration of different formulations able to provide a safe injection method, we got the desired concentrations using a vehicle of ethanol, polyethylene glycol 400, and saline (1:300:200, *v/v*). Using this vehicle, animals were treated intravenously with QN23 concentrations of 1.0, 1.5, 2.0, and 2.5 mg/kg as a single injection at the onset of the reperfusion period after transient cerebral ischemia. After five days of recovery (R5d), neuronal death, apoptosis, and neurodeficit score were determined (Figure 1 and Figure 2).

#### 3.1.1. Analysis of Neuronal Death

Interruption of blood supply to the brain for 15 min followed by reperfusion induced neuronal death, especially in the hippocampal CA1 and cortical regions of the brain, which could be observed by Fluoro-Jade B staining after 5 days of reperfusion. In the experiments summarized in Figure 1, fewer dead neurons were observed after QN23 treatment than after treatment with vehicle in the entire concentration range analyzed, both in the hippocampal CA1 region and cerebral cortex (Figure 1a,b). In particular, when treated with 2.0 or 2.5 mg/kg of QN23, the neuronal death was significantly reduced in the hippocampal CA1, whereas in the cerebral cortex it was when treated with 2.0 mg/kg of QN23. Expressing the induced neuroprotection as a percentage, and considering the number of dead neurons present in the vehicle group as 0% neuroprotection, a dramatic increase in neuroprotection could be observed for QN23 at 1.5 and 2.0 mg/kg in the cortical region, with this effect being less sharp in the hippocampal CA1 region (Figure 1c).

#### 3.1.2. Analysis of Neuronal Apoptosis

In addition to neuronal death due to the ischemic–reperfusion damage, triggering of the ischemic cascade activated apoptosis mechanisms within the neuronal tissue. As a consequence of a 15 min period of cerebral ischemia, neuronal apoptosis was observed in the brains after five days of post-ischemic reperfusion by TUNEL assay, as explained in the Materials and Methods section. The results shown (Figure 1d,e) describe the cell apoptosis reduction observed when animals were treated with QN23 (range 1.0–2.5 mg/kg) compared to the vehicle group. In accordance with the neuronal death determination by Fluoro-Jade B experiments, a significant decrease in the number of apoptotic cells was obtained with QN23 at 2.0 and 2.5 mg/kg in both CA1 and cortical regions when compared to vehicle. When expressed as neuroprotection percentage, a notable increase in neuroprotection was observed for QN23 in the cortical region, with lesser effect in the hippocampal CA1 region (Figure 1f) (see below).

#### 3.1.3. Analysis of Neurodeficit Score

For the quantification of a potential functional impairment of the animals subjected to the ischemic–reperfusion insult and treatment, their general status was assessed as described in the Materials and Methods section. In this test, affection on different variables related to the status of the animal (breathing, aspect, coordination, etc.) is quantified by numerical rating. The total rating accounts for the neurological deficit score (NDS) of the animal, which describes its general consciousness and neurological activity, ranging from 0 (healthy animal) to 10 (animal with a severely decreased level of consciousness and impaired neurological activity). As shown in Figure 2, a significant improvement of the general status of the animal (i.e., a reduced NDS) compared to vehicle was observed when QN23 was administered at a dose of 2.0 mg/kg.

#### 3.1.4. Dose–Response Curve

The observed decrease in the number of dead neurons and apoptosis-positive cells in the Fluoro-Jade B and TUNEL assays, respectively, indicated a neuroprotective effect of QN23 in the experimental model used in this study. These results, expressed as a percentage, in which the number of dead neurons and apoptotic cells observed for the vehicle-treated group accounts for 0% of neuroprotection and 100% stands for a complete absence of neuronal death and apoptosis, are represented in Figure 1c (neuroprotection in neuronal death studies) and Figure 1f (neuroprotection in apoptosis studies), respectively. As described in these graphs, in the neuronal death results maximum neuroprotection was achieved at doses of 1.5 and 2.0 mg/kg, both in the hippocampal CA1 region (22.6 and 23.8%, respectively) and in the cerebral cortex (89.2 and 89.5%, respectively), as well as in the neuronal apoptosis results in the CA1 (16.2 and 27.8%, respectively) and cortical (85.0 and 88.0%, respectively) regions. These results prompted the selection of 2.0 mg/kg as the optimal dose of QN23 for the following studies. The determination of the EC_50_ based on this representation gave a value of 0.3 mg/kg either in the CA1 or in cortical regions, and both for neuronal death and TUNEL assays.

### 3.2. In Vivo Antioxidant Capacity of QN23

**Figure 3:**
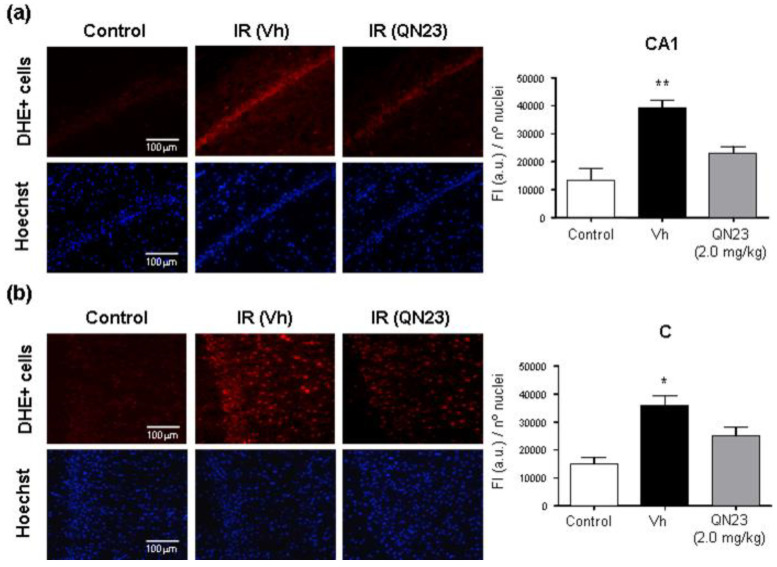
Antioxidant capacity of QN23 in vivo. Ischemic animals were treated with QN23 (2.0 mg/kg) or vehicle (Vh) by intravenous injection at the onset of reperfusion after cerebral ischemia (IR), dihydroethidium (DHE, 3.0 mg/kg) was injected intravenously at 2 h after reperfusion, and the animals were euthanized at 4 h after DHE injection. Control animals were also injected with DHE and euthanized 4 h after injection. The antioxidant effect of QN23 was studied by quantification of fluorescence intensity of ROS-reacted DHE in brain sections. Representative images of cells stained with ROS-reacted DHE (DHE+ cells) in the hippocampal CA1 (**a**) and cortical (**b**) regions. Bar graphs represent the fluorescence intensity (FI) quantified in brain sections from control, vehicle-, and QN23-treated animals injected with DHE in the hippocampal CA1 (CA1) and cortical (C) regions. Data represent the mean of 3–5 independent animals per group (3 control animals, 5 animals treated with vehicle, and 4 treated with QN23); error bars indicate the SE. * *p* < 0.05 and ** *p* < 0.01, compared with the control or QN23-treated group by Newman–Keuls’ post-test after ANOVA. FI is expressed in arbitrary units (a.u.).

Antioxidant capacity of QN23 was previously assayed in vitro and in primary neuronal cultures subjected to OGD as an experimental model of hypoxia. It was discovered that QN23 behaved as a potent scavenger of hydroxyl radical (·OH), and that it decreased ROS and lipid peroxidation levels [22]. In this experiment we studied the antioxidant capacity of QN23 in vivo using dihydroethidium (DHE) as a fluorescent probe sensitive to superoxide and hydrogen peroxide radical species. DHE was injected 2 h after QN23 or vehicle treatment in animals previously subjected to cerebral ischemia (Figure 3). A control experimental group, with animals without the surgical procedure to induce ischemia, was included for comparative purposes. We used the most effective dose (2.0 mg/kg) found after the dose–response studies (see Results, Section 3.1).

The results presented in Figure 3 show that, as a consequence of the transient ischemic episode, a significant increase in the fluorescence intensity, meaning a higher production of radical species, was observed in the vehicle-treated group compared to the basal control group (2.9- and 2.4-fold increase in the hippocampal CA1 and cortical region, respectively). When the animals were treated with QN23 2.0 mg/kg, the fluorescence intensity of ROS-reacted DHE decreased significantly compared with the vehicle-treated group, being slightly higher than the control group but with no statistical difference. The increase in the fluorescence intensity—i.e., increased ROS levels—due to the experimental ischemia was observed in the CA1 and cortical regions, and in both regions QN23 significantly decreased the fluorescence intensity rise. Control experiments were carried out as described in Figure 3, injecting fluorescent oxidized DHE to verify that the permeability of DHE into the brain was not different between control and ischemic animals (results not shown).

### 3.3. Therapeutic Window

In order to explore the feasibility of a delayed administration of QN23, functional and neuroprotection assessments were performed on animals subjected to cerebral ischemia and subsequent reperfusion (see Materials and Methods, Section 2.2) by administering QN23 at time points ranging from 0 to 6 h after the reperfusion onset. QN23 was administered as a single intravenous injection at 2.0 mg/kg as the most effective dose found in the dose–response study (see above). Reference compound NXY-059 (40 mg/kg) was also included in this study for comparative purposes. The dose of NXY-059 (40 mg/kg) was calculated as the average dose among the doses assayed in rat experimental ischemia models for neuroprotection (doses ranging from 0.3 to 120 mg/kg) [10]. The neurodeficit score was evaluated in animals after five days of reperfusion, along with neuronal death and apoptosis, in the excised brains as described in the Materials and Methods section.

#### 3.3.1. Analysis of Neuronal Death

**Figure 4:**
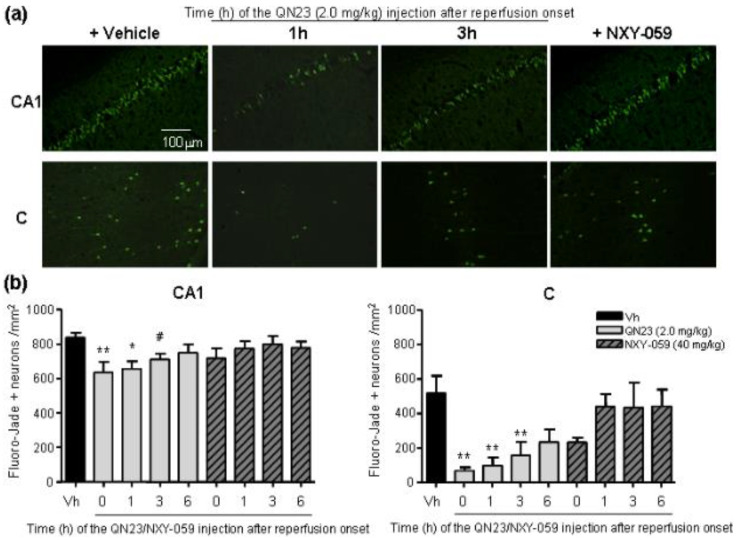
Therapeutic window of QN23 studied by Fluoro-Jade B assay (neuronal death). Ischemic animals were treated with vehicle (Vh), QN23 (2.0 mg/kg), or NXY-059 (40 mg/kg) by an intravenous injection at reperfusion onset (0 h), or at 1, 3, or 6 h of reperfusion after global cerebral ischemia. Neuroprotection was evaluated after 5 days of post-ischemic reperfusion. (**a**) Representative images of Fluoro-Jade B-stained hippocampal CA1 (CA1) and cortical (C) brain sections of vehicle- and QN23-treated animals at 1 h or 3 h of reperfusion and NXY-059-treated animals at 0 h of reperfusion. Dead neurons observed after Fluoro-Jade B staining within the CA1 and C regions were imaged by fluorescence microscopy (in green) and counted as described in the Materials and Methods section. (**b**) Bar graphs represent the neuronal death quantified in brain sections from vehicle-, QN23-, and NXY-059-treated animals in CA1 and C regions. For each group, 6–10 independent animals were averaged (10 animals treated with vehicle, 6–7 animals per QN23 treatment, and 6 animals per NXY-059 treatment); error bars indicate the SE. * *p* < 0.05 and ** *p* < 0.01, compared with vehicle (Vh) by Dunnett’s post-test, and # *p* < 0.05, by Student’s *t* test, after ANOVA.

Administration of QN23 at a dose of 2.0 mg/kg significantly decreased neuronal death both in the hippocampal CA1 and cortical regions when injected up to 3 h of post-ischemic reperfusion, as observed with Fluoro-Jade B staining (Figure 4), compared to the vehicle group. In contrast, reference nitrone NXY-059 (40 mg/kg) administration did not reduce neuronal death compared to vehicle at any of the time points studied.

#### 3.3.2. Analysis of Neuronal Apoptosis

**Figure 5:**
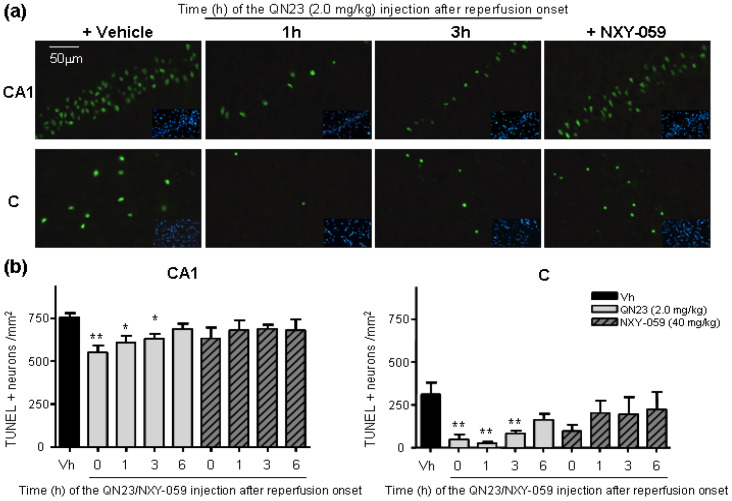
Therapeutic window of QN23 studied by TUNEL assay (apoptosis). Brain sections of ischemic animals from Figure 4 were used for apoptosis detection by TUNEL assay in the hippocampal CA1 (CA1) and cortical (C) regions and imaged by fluorescence microscopy (in green). (**a**) Representative images of CA1 or C regions from vehicle- and QN23-treated animals at 1 h or 3 h of reperfusion and NXY-059-treated animals at 0 h of reperfusion, after apoptosis detection by TUNEL assay and the corresponding Hoechst counterstaining. TUNEL-positive cells detected within CA1 and C regions were counted as described in the Materials and Methods section. (**b**) Bar graph representations of TUNEL-positive cells quantified in brain sections from vehicle-, QN23-, and NXY-059-treated animals in CA1 and C regions. For each group, 6–10 independent animals (as described in Figure 4) were averaged; error bars indicate the SE. * *p* < 0.05 and ** *p* < 0.01, compared with vehicle (Vh) by Dunnett’s post-test, after ANOVA.

The results obtained in the evaluation of neuronal apoptosis (Figure 5) showed a pattern similar to that obtained in the neuronal death analysis. Administration of QN23 at a dose of 2.0 mg/kg at extended periods after the onset of reperfusion significantly reduced the number of apoptosis-positive cells when injected up to 3 h in both CA1 and cortical regions compared to the vehicle group. Again, NXY-059 40 mg/kg did not produce a significant effect on apoptosis reduction compared to the vehicle group at the administration time points studied.

#### 3.3.3. Functional Test

**Figure 6:**
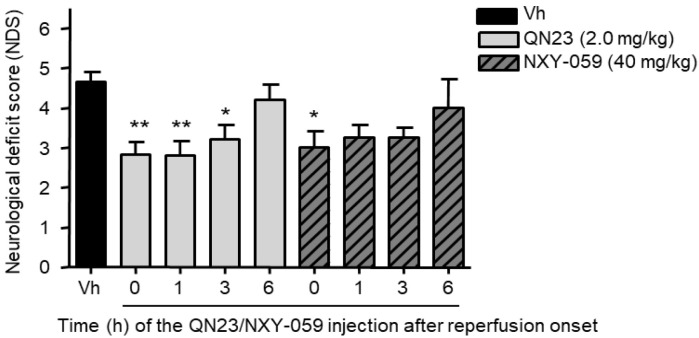
Neurodeficit score outcomes after cerebral ischemia in the therapeutic window study of QN23. The bar graph shows the neurological deficit score (NDS) in ischemic animals after 5 days of reperfusion after ischemia. Animals were treated with QN23 (2.0 mg/kg), NXY-059 (40 mg/kg), or vehicle (Vh) by intravenous injection at the reperfusion onset (0 h), or after 1, 3, or 6 h of post-ischemic reperfusion. For each group, 6–10 independent animals (as described in Figure 4) were averaged; error bars indicate the SE, * *p* < 0.05 and ** *p* < 0.01 compared with vehicle by Dunn’s post-test after Kruskal–Wallis test.

The functional assessment of animals injected at different time points was determined by a general status evaluation using a neurological deficit score (NDS). As shown in Figure 6, a significant improvement in the general status of the animal (i.e., a reduced NDS) compared to vehicle was observed when QN23 was administered up to 3 h after the onset of reperfusion, improvement that was only observed with NXY-059 treatment at the reperfusion onset (0 h).

### 3.4. Long-Term Efficacy Assessment

Considering the relevance of a long-term effect in stroke therapeutics, our next objective was to assess the effect of the optimal dose of QN23 found in previous short-term studies (5 days; 2.0 mg/kg) at longer times after the ischemic episode. This approach, however, requires alternative methods to the Fluoro-Jade B staining or TUNEL assay described before, since damaged and dead cells are naturally removed by endogenous clearance systems and, therefore, not observed at longer times with these assays. For this purpose, and as reported previously [18], we analyzed, by immunostaining, the number of viable cells positive to ribosomal protein S6, a constitutive protein present in the neuron cytoplasm and marker of cell viability [34]. With this approach, we determined the neuronal viability within the hippocampal CA1 region at 11 weeks of reperfusion after the ischemic episode by comparison with whole neurons in sections from control animals. Ischemic animals (see Materials and Methods, Section 2.2) were treated with vehicle, QN23 (2.0 mg/kg), or NXY-059 (40 mg/kg) at the onset of reperfusion. Animal behavior was also analyzed to assess long-term neurological sequelae, as described in the Materials and Methods section.

#### 3.4.1. Analysis of Neuronal Viability

**Figure 7:**
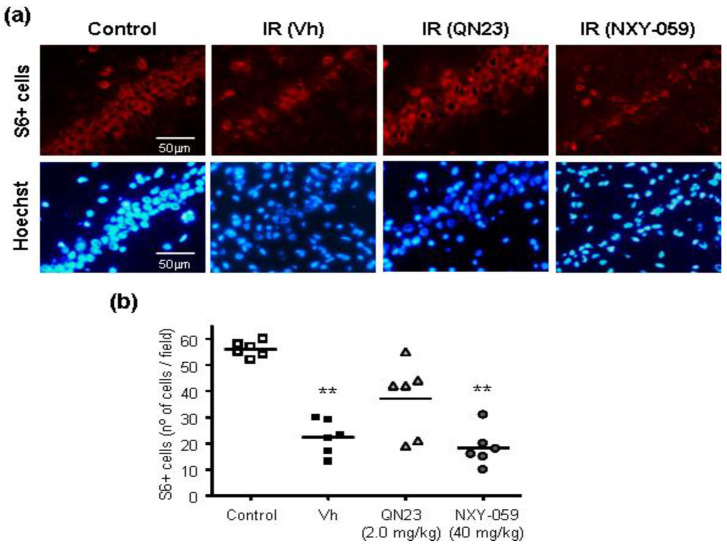
Study of the long-term effect of QN23 after cerebral ischemia–reperfusion by neuronal viability assessment. Brain sections from control animals (control) or from animals treated with vehicle (Vh), QN23 (2.0 mg/kg), or NXY-059 (40 mg/kg) by intravenous injection at the reperfusion onset after ischemia were evaluated for immunostaining at 11 weeks after ischemic reperfusion (IR). Neurons were labeled with anti-S6 protein antibody and X Red-secondary antibody and visualized by fluorescence microscopy (in red). Cell nuclei were counterstained with Hoechst dye (in blue). (**a**) Representative images of the hippocampal CA1 region. (**b**) Dot graph representation of S6-labeled cells (S6+ cells) per field of the hippocampal CA1 region from control animals and vehicle (Vh)-, QN23-, and NXY-059-treated animals after 11 weeks of post-ischemic reperfusion. Individual values of 6 independent animals per group are shown and the horizontal line represents the mean. ** *p* < 0.01, compared with the control group by Dunnett’s post-test after ANOVA.

S6 protein immunostaining was performed in brain sections from control or ischemic-reperfused animals for 11 weeks and observed in the CA1 region of the hippocampus. Viable neurons were stained by S6, and its number was compared between the different experimental groups (control, vehicle, QN23, or NXY-059 (Figure 7)). As shown in the figure and the corresponding data quantification graph, fewer S6-positive cells (i.e., viable neurons) were observed in ischemic animals treated with vehicle solution (Figure 7a, IR Vh) compared to control animals. These results show how 15 min of cerebral ischemia followed by reperfusion damaged neuronal cells within the CA1 region of the hippocampus—the cerebral region involved in short-term memory and spatial memory storage—and that even 11 weeks of recovery after reperfusion failed to overcome this situation, inducing irreparable damage to an essential brain structure. Remarkably, treatment with NXY-059 (Figure 7a, IR NXY-059) did not avoid this issue, with a similar number of positive cells observed as in the vehicle group (Figure 7b). In contrast, treatment with QN23 (Figure 7a, IR QN23) preserved a large number of viable neurons compared to the control group, with no significant differences between these two groups (Figure 7b). In addition, the morphology and disposition of the neurons were similar to those of control animals, in what represents a long-term effect of QN23 in the structural integrity preservation of the hippocampal CA1.

#### 3.4.2. Behavioral Tests

The neurodeficit score determination described above is not suitable for a long-term assessment of potential neurological deficits. Due to the inherent plasticity of the nervous system, animals may overcome the functional deficits appearing shortly after the ischemic episode, recovering completely several days after ischemia and not being observable with a general status measurement such as NDS. For this reason, behavioral tests assessing potential sequelae present in the long term after the ischemic reperfusion period, such as those assessing spatial recognition, memory, orientation, and curiosity, for instance, are required. In these experiments, we conducted two different behavioral tests to evaluate the spatial recognition and spatial memory of the animal in order to determine potential long-term cognitive impairment (see the Materials and Methods section for detailed procedure information).

##### Exploratory Activity and Spatial Recognition Test

**Figure 8:**
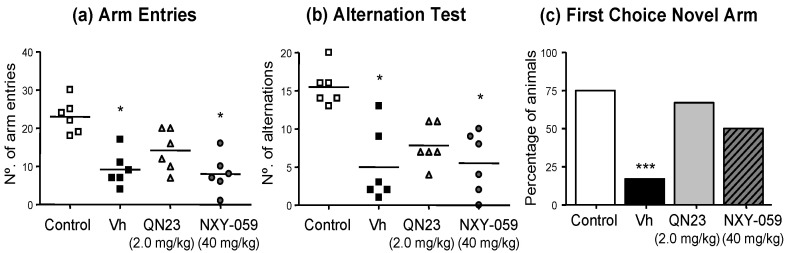
Study of the long-term effect of QN23 after cerebral ischemia–reperfusion by behavioral tests. Graphic representations of the results of three tests assessing the spatial recognition and spatial memory, as long-term cognitive sequelae, of control, vehicle-, QN23-, and NXY-059-treated animals 11 weeks after post-ischemic reperfusion. In (**a**,**b**), individual values of 6 independent animals per group are shown and the horizontal line represents the mean. * *p* < 0.05, compared with the control group by Dunn’s post-test after the non-parametric Kruskal–Wallis test, *** *p* < 0.001, compared with the vehicle (Vh) group by Fisher’s test. In (**c**), two animals of the control group had a null test.

For the assessment of the exploration activity, vehicle-, QN23-, and NXY-059-treated animals were compared to a control group. As shown in Figure 8a, animals from the vehicle group showed fewer arm maze entries, in a similar number to the animals treated with NXY-059 compared to control. In contrast, animals treated with QN23 showed a higher number of new entries (differences not significant compared with control), accounting for a preserved neurological function.

Also in this test, spatial recognition was assessed by the number of alternations or times that the animals completed a triad (i.e., three different arms of the maze consecutively), as can be seen in Figure 8b. As for the arm entry evaluation, the vehicle group completed fewer triads (5.0) than the animals from the control group (15.75). Again, treatment with NXY-059 did not produce better performance in this test (5.5), whereas animals treated with QN23 improved their score (7.8) and showed no significant differences compared to the control group.

##### Spatial Memory Test

A long-term spatial memory assessment was conducted by a novel arm recognition test, also in the Y maze. Briefly, after a training period with one of the arms closed, the animals were placed again in the same starting arm of the maze with the other two arms available for exploration. The exploration of the new arm as a first choice is indicative of proper spatial memory and, therefore, was assessed for control, vehicle-, QN23-, and NXY-059-treated animals. The data obtained (Figure 8c) showed a decreased percentage of animals within the vehicle group exploring the new arm as a first choice (17% compared with 75% of the control, *p* < 0.0001), indicating impaired spatial memory. In contrast, treatment with QN23 showed an increased percentage of animals exploring the novel arm as a first option, with values similar to the control group (67%) and with no significant difference between these two groups (*p* = 0.1376). Contrary to previous tests, animals treated with NXY-059 also showed an increased percentage (50%), although less than the QN23-treated group.

### 3.5. QN23 Treatment in Transient Focal Cerebral Ischemia Induced by Intraluminal Suture Procedure: Dose–Response Study

The cerebroprotective effect of QN23 on brain damage induced by ischemic stroke was assessed in a rat model of tMCAO. The reduction of cortical perfusion during ischemia was comparable in all the experimental groups, as was the extent of perfusion increase during reperfusion. Mean arterial blood pressure, core temperature, and serum glucose were in the physiological range and similar in all groups.

**Figure 9:**
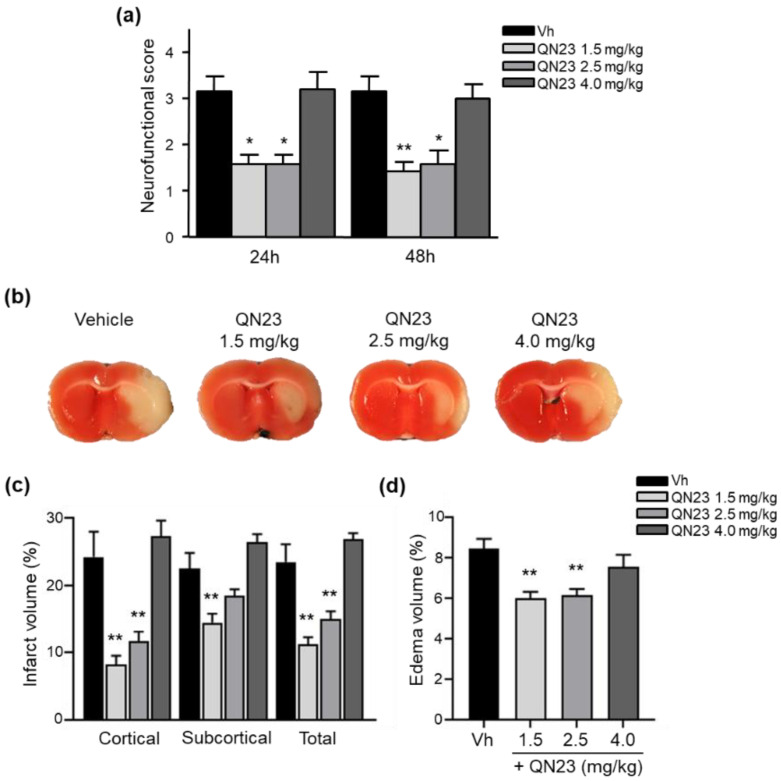
Cerebroprotective effects of QN23 in male Wistar rats subjected to ischemic stroke and treated with vehicle (Vh, *n* = 7); QN23, 1.5 mg/kg (*n* = 7); QN23, 2.5 mg/kg (*n* = 7); and QN23 4.0 mg/kg (*n* = 5). (**a**) Neurofunctional score at 24 h and 48 h after tMCAO (transient middle cerebral artery occlusion). (**b**) Representative images of 2 mm-thick coronal sections (0.2 to −1.8 mm from bregma) stained with 2,3,5-triphenyltetrazolium chloride (TTC) to show the infarcted (pale) area at 48 h after tMCAO. (**c**) Cortical, subcortical, and total infarct volumes at 48 h after tMCAO. (**d**) Edema volume at 48 h after tMCAO. Statistical significance compared with vehicle, * *p* < 0.05 and ** *p* < 0.01, was done between vehicle and 1.5 mg/kg and 2.5 mg/kg QN23-treated groups by Dunn’s post-test after the non-parametric Kruskal–Wallis test in (**a**), and by Dunnett’s post-test after ANOVA in (**c**,**d**).

Intravenous treatment with QN23 at 1.5 mg/kg and 2.5 mg/kg, but not at 4 mg/kg, significantly reduced ischemia/reperfusion-induced neurofunctional impairment at both 24 h and 48 h after the ischemic insult compared with the vehicle treatment (Figure 9a). Of note, four rats in the QN23 4 mg/kg group died before 24 h. The filament tMCAO model resulted in an infarct lesion involving both the cortical and the subcortical region of the MCA territory (Figure 9b). In line with the neurofunctional improvement, QN23 at 1.5 mg/kg and 2.5 mg/kg, but not at 4 mg/kg, significantly reduced brain damage assessed at 48 h compared with vehicle treatment. Both total infarct and edema volumes were reduced (Figure 9c,d, respectively). Infarct reduction was higher at the cortical than at the subcortical level (Figure 9c).

### 3.6. Toxicokinetics Study

**Figure 10:**
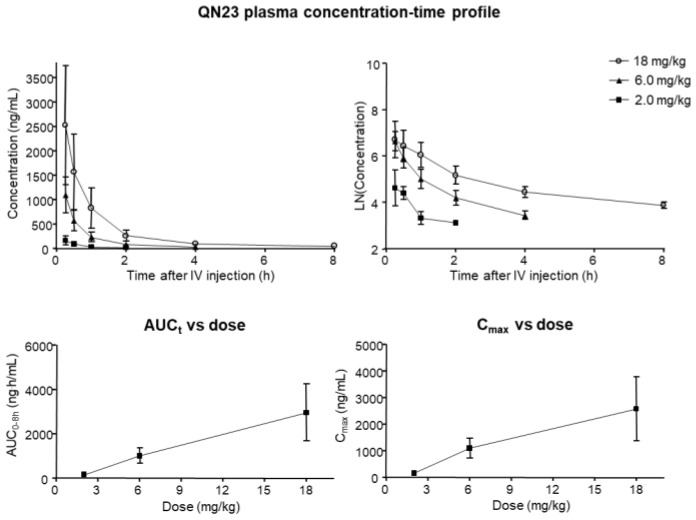
QN23 plasma concentration–time profile after intravenous administration and values of AUC_0–8h_ (AUC_t_) and C_max_ versus dose. Data represented as mean ± SE of 6 independent animals. AUC, area under the curve.

A study of the toxicokinetics of QN23 was carried out by determining the concentration of QN23 in plasma after a single bolus intravenous injection of our working dose of 2.0 mg/kg, and higher doses of 6.0 and 18 mg/kg. In these assays, a vehicle solution with a higher concentration of polyethylene glycol 400 was necessary for the solubilization of the elevated doses of QN23 tested. Plasma samples were collected 0.25, 0.5, 1, 2, 4, and 8 h after administration, and QN23 concentration was determined by LC/MS-MS (Figure 10). A non-compartmental analysis was used for the calculation of the pharmacokinetic parameters (Table 1).

QN23 was detected in blood after intravenous administration, and its concentration declined progressively for the three doses studied (Figure 10). In all cases, the rate of concentration decrease was very fast, with very short half-lives ranging from 0.16 h (2.0 mg/kg) to 1.13 h (18 mg/kg). Concentration values of animals treated with 2.0 mg/kg QN23 reached the limit of quantification between 2 and 4 h, and animals treated with 6 mg/kg QN23 showed no quantifiable concentration later than 4 h after injection. When QN23 was administered at 18 mg/kg, the concentration found (49 ± 5 ng/mL) represented approximately 1% of the initial (and maximal) detected concentration. These results show that the parameter AUC_0–8h_ reflects the total exposure of QN23 as a single bolus administration, and increased proportional to the dose administered (AUC vs dose). Noteworthy, there was a high variability between animals, especially for higher doses.

Regarding the toxicity in a four-day repeated-dose study of QN23, no treatment-related systemic signs were detected after intravenous administration of QN23 at any of the three dose levels tested (2.0, 6.0, and 18 mg/kg) compared with the control group. Only hematoma formation over the injection site was observed in all study groups from day 2 of treatment, including the control group, an effect that may be considered related to the vehicle or the administration procedure itself. Body weight loss, which ranged from 5.28 to 8.23%, was observed and was of similar magnitude in all groups, including control animals, and no effects were observed in food intake. In the necropsies collected (heart, kidneys, liver, and spleen) upon completion of the treatment period, no macroscopic findings for any of the organs evaluated were observed in any of the animals dosed, and no significant differences in absolute or relative organ weights were observed between QN23-treated groups and the control group. Clinical biochemistry parameters in blood samples were determined and are included in the Supplementary Material (Table S1). Only slight variations in alkaline phosphatase (ALP, decreased) and aspartate aminotransferase (AST, increased) levels were found at some doses of QN23, but no treatment-related effects were concluded. Taken together, the results suggest that it can be concluded that the repeated intravenous administration of QN23 for four days at dose levels of 2.0, 6.0, and 18 mg/kg was well tolerated, since no systemic toxic effects were observed.

## 4. Discussion

In the current clinical practice, there is a direct need for therapies for the treatment of ischemic stroke. Recanalization strategies, either mechanical (thrombectomy) or pharmacological (e.g., rtPA), are the only therapies approved by the Food and Drug Administration or European Medicines Agency for the treatment of this pathology and must be combined with new approaches in order to effectively improve the patient’s recovery and decrease damage associated with ischemic reperfusion injury. In this paper, we updated the assessment of the biological effect of QN23 as a preclinical candidate for the treatment of ischemic stroke by reporting, based on different animal experimental models, a dose–response study, in addition to a therapeutic window screening and an evaluation of the long-term efficacy. Remarkably, we include here the in vivo antioxidant activity and also report the results of a pharmacokinetics study of QN23.

In this report, the administration of QN23 was determined to be feasible, safe, and effective as an intravenous single injection, and therefore, potentially useful for the treatment of pathologies such as acute ischemic stroke (AIS). The solubility of the compound required of an in-house designed vehicle was composed of ethanol, polyethyleneglycol 400, and saline, which was proven safe in healthy animals (data not shown). With this approach, doses ranging from 1.0 to 2.5 mg/kg was assayed on a transient four-vessel occlusion (4VO) model, and the effect, based on the evaluation of neuronal death and apoptosis induction, concluded 2.0 mg/kg as the most effective dose. An independent laboratory performed the study of the neuroprotective effect of QN23 on a transient middle cerebral artery occlusion (tMCAO) model by intraluminal suture procedure, a preclinical relevant model of focal ischemia. Dose–response analysis included 1.5, 2.5, and 4.0 mg/kg. Evaluation of the functional outcomes was in accordance with the infarct and edema volumes measured on the ischemic brains of the animals and reported the range of 1.5–2.5 mg/kg as the most effective. These results agree with the optimal dose found for the global ischemia model. The aforementioned limited solubility of QN23 in aqueous solutions may explain the lower effect with high doses of QN23, since it is feasible that with QN23 at the high doses used, part of the compound may have precipitated as an insoluble compound after the injection, and therefore been ineffective. This effect could be more intense at the highest dose used. Of note, four animals treated with QN23 4.0 mg/kg died after the administration within the next 24 h, in contrast with one animal death in the group treated with vehicle and QN23 2.5 mg/kg. Given that the toxicokinetic study tested higher doses of QN23 (6.0 and 18 mg/kg) without finding apparent effects of toxicity, the deaths may have been caused by a poor solubility and/or precipitation of the compound in a vehicle solution with lower polyethylene glycol 400 concentration than in the toxicokinetic study, causing the obstruction of a vessel and a lack of effectiveness.

As radical traps, nitrones such as QN23 have long been thought to exert their neuroprotective mechanism of action by stabilizing highly reactive radical species and avoiding further damage to the cell structures [9]. Previous studies of the antioxidant activity of QN23 revealed a very potent scavenging activity against hydroxyl radical, the most reactive and harmful radical species produced during ischemia–reperfusion damage [22]. Furthermore, QN23 was reported to significantly reduce ROS and lipid peroxidation levels in primary neuronal cultures subjected to OGD to comparable levels of undamaged cells [22]. However, could this activity be translated to in vivo? There has been controversy about the real effect of radical scavengers in vivo, raised also for nitrones such as PBN or NXY-059, mainly due to the unachievable high concentrations required in cerebral tissue in order to effectively act as radical traps [9]. In this report we describe for the first time evidence of the antioxidant effect of QN23 in vivo, using dihydroethidium (DHE) as a molecular probe for oxidative stress [29,35]. The obtained results show a remarkable effect of QN23 on the suppression of the fluorescence intensity of ROS-reacted DHE, which accounts for a decreased production of radical species and meaning that QN23 may indeed work as an antioxidant nitrone. Based on the extensive literature of nitrones working as radical traps, it seems coherent to attribute this effect to radical-trapping activity. However, other mechanisms different to radical trapping, such as an activation of endogenous antioxidant systems, may also participate in this reduction in ROS-reacted DHE fluorescence intensity [36]. In any case, the results reported here shed light on a controversial field such as the in vivo effect of an antioxidant, which was proven effective in this report.

All of the previous studies were carried out with QN23 being administered at the onset of reperfusion. This served as a way to find the most effective dose of QN23 in our experimental model and to determine an antioxidant mechanism of action of QN23, but this injection schedule is less likely to occur in real clinical practice. After the first signs of an acute ischemic attack, the management, diagnosis, and treatment of a stroke patient may take several hours and, therefore, any drug candidate must be proven safe and effective also at administration times with relatively long delays from symptom onset and/or recanalization therapy, mirroring clinical practice in stroke patients [3]. In this report we explored a delayed time of injection ranging from 1 to 6 h post ischemia. Significant reduction of damage outcomes were observed with QN23 (2.0 mg/kg) injection up until 3 h (R3h) in our preclinical model, with an effect comparable to the one observed when treatment was administered just after the ischemic period. Remarkably, treatment with NXY-059 (40 mg/kg) also produced a slight improvement, although not significant, with the same administration schedule. Whether this extended time of administration might be applicable to humans and compatible with current recanalization approaches must still be assessed, but it represents an interesting starting point into the development of more clinically useful therapies.

Related to this, the influence of an extended therapeutic window on the mechanism of action of QN23 is an aspect that still needs to be assessed. Considering the antioxidant activity of QN23 as the responsible mechanism for the neuroprotective effect found, an extended administration time, later than the peak of radical species usually produced right after the recovery of normal oxygen levels in the compromised tissue, may explain the decrease in the effectivity in our experimental model. In other words, we can consider that the later QN23 is injected, the less effective it is at decreasing radical species levels, since they have already been generated and damage to cellular structures has already been produced. This may justify that the antioxidant activity of QN23 is its main beneficious role, and that further mechanisms of action—i.e., binding to biological targets, modulating signaling pathways, etc.—that could take place at later times (i.e., at 6 h) would not be expected to have a crucial influence on the final outcome.

Long-term sequelae of ischemic stroke, manifested normally as psychiatric or neurological disorders, represent an aspect that is gaining importance in the current clinical practice and social security systems due to an increased incidence of stroke episodes in younger adults. The evaluation of a potential protective effect longer term after an ischemic episode is, therefore, of crucial relevance. The use of preclinical models to assess this kind of outcome may be problematic since life span comparison between species is hard to evaluate. Some literature has mirrored a human life period of five to seven years to a period of three months in adult rats [37]. Regardless of the exact equivalence between the two species, this time period allows for the determination of outcomes, either functional or biochemical, once the brain-remodeling action after the ischemic–reperfusion injury carried out by endogenous neurorepair processes has been completed [38]. The immunofluorescence detection of ribosomal protein S6 after 11 weeks (three months) of reperfusion allowed for the observation of the anatomical disruption produced within the hippocampal CA1 region as a consequence of the ischemic-reperfusion injury. These results were confirmed by behavioral tests, which detected an impaired performance in the spontaneous spatial recognition and spatial memory tests in those untreated ischemic animals. Remarkably, treatment with QN23 decreased the long-term functional impairment and preserved neuronal viability at a three-month-long period, which adds to the short-term effect described previously and contributes to the preclinical confirmation of QN23 as a promising candidate for the treatment of ischemic stroke.

As its name indicates, acute ischemic stroke is characterized by a sudden appearance of symptoms, and the therapeutic window, spanning only several hours, requires a rapid and efficient route of administration. A single bolus intravenous injection allows for a fast delivery of drugs to the systemic circulation, rapidly reaching the target tissue, i.e., the brain. Determination of the characteristics of this type of administration for QN23 in terms of the achieved concentration, time of elimination or accumulation, and potential toxicity, allows for the optimization of a dose regime for further development. In this report, we analyzed the pharmacokinetics parameters of different doses of QN23 (2.0, 6.0, and 18 mg/kg) when injected intravenously as a single bolus in healthy animals in a period of 8 h after administration. Non-compartmental analysis of the obtained concentration–time profile showed a very fast and gradual decrease in concentration in blood in the time studied, with very short half-lives ranging from 0.16 (2.0 mg/kg) to 0.24 h (6.0 mg/kg) or 1.13 h (18 mg/kg), and rapid elimination due to high clearance rates. This fast elimination of QN23 from the organism may indicate a very fast mechanism of action able to produce the effect described in this report in a period of minutes but, at the same time, with consequences long after the injection, as described in the long-term-effect assessment. Regarding the relevance of oxidative stress in the course of tissue recovery after ischemia–reperfusion, this fast mechanism of action of QN23 agrees with the evidence demonstrated here that the antioxidant activity of QN23 would be its main target of action.

Finally, this study was designed and conducted with predefined exclusion criteria, physiological monitoring, sample randomization, allocation concealment, blinded assessment of outcomes, dose–response definition, and a conflict-of-interest statement, in accordance with STAIR and RIGOR guidelines. In addition, we assessed preclinical relevant aspects of the potential use of QN23 as a treatment, such as its therapeutic window, its long-term effect, and its pharmacokinetics, including preliminary results on its antioxidant mechanism of action [3,17]. For these studies we used a four-vessel occlusion (4VO) global ischemia and a transient middle cerebral artery occlusion (tMCAO) focal ischemia as preclinical relevant models for preclinical studies. As a first preclinical characterization, we decided to use young-adult male rats in order to assess important features of QN23 performance and the mechanism of action in more standardized systems. The results obtained in these models are indispensable in preliminary steps of preclinical characterization, and these results are required for design optimization in more advanced preclinical stages.

## 5. Conclusions

In this report we updated the efficacy and characterization of QN23 as a potential therapeutic agent for the treatment of acute ischemic stroke. This molecule may have a prominent therapeutic interest in combination with mechanical thrombectomy or thrombolytic agents to ameliorate the reoxygenation-induced injury after the induced reperfusion. In addition, we showed evidence of its mechanism of action, pharmacokinetic properties, and toxicity, as a preliminary pharmacological characterization. New compounds that will be initiated in clinical trials for the treatment of ischemic stroke require a complete characterization during preclinical phases, addressing key issues for compound efficacy and safety. Our results for QN23 encourage the continuation of preclinical studies with this potential drug.

## 6. Patents

Alberto Alcázar González, Emma Martínez Alonso, José Luis Marco Contelles, and Mourad Chioua Asri. Quinolylnitrones for the treatment and prevention of a cerebral stroke or ischaemia. Application PCT/EP2019/077525.

## Figures and Tables

**Table 1:** Pharmacokinetic parameters of QN23.

Dose (mg/kg)	C_0_ ^1^ (ng/mL)	C_max_ (ng/mL)	AUC_0–8h_ (ng·h/mL)	C_last_ (ng/mL)	V_d_ (mL)	k_el_ (h^−1^)	Cl (mL/h)	t_1/2_ (h)
2.0	540 ± 186	161 ± 86	160 ± 61	30 ± 8	1261 ± 434	4.24 ± 0.38	4400 ± 1685	0.16 ± 0.01
6.0	2172 ± 661	1094 ± 371	1012 ± 355	34 ± 5	1657 ± 584	3.35 ± 0.57	3402 ± 1559	0.24 ± 0.03
18	4350 ± 1861	2571 ± 1202	2958 ± 1287	49 ± 5	20435 ± 12334	1.25 ± 0.42	4685 ± 1621	1.13 ± 0.39

^1^ C_0_: initial concentration; C_max_, maximal concentration; AUC_0–8h_: area under the curve for the period of 0–8 h; V_d_: volume of distribution; K_el_: elimination constant; Cl, clearance; t_1/2_, half-life. Data as mean ± SE of 6 independent animals.

## Data Availability

The data presented in this study are available in the article.

## Supplementary Materials

The following are available online at https://www.mdpi.com/article/10.3390/antiox11061186/s1, A. Toxicokinetics study. Clinical biochemistry parameters (Table S1).
