# Supplementary material for: Preclinical Characterization of Antioxidant Quinolyl Nitrone QN23 as a New Candidate for the Treatment of Ischemic Stroke
Source: Antioxidants (Basel). 2022 Jun 16;11(6):1186. doi: 10.3390/antiox11061186 (PMC9220178; doi:10.3390/antiox11061186)
Supplement: Supplementary file 1 [file antioxidants-11-01186-s001.zip › antioxidants-1729634-supplementary.pdf]

Supplementary Material

Preclinical Characterization of Antioxidant Quinolyl Nitrone QN23 as a New Candidate for the Treatment of Ischemic Stroke

Emma Martínez-Alonso [1], Alejandro Escobar-Peso [1], Alicia Aliena-Valero [2], Germán Torregrosa [2], Mourad Chioua [3], Rocío Fernández-Serra [4], Daniel González-Nieto [4,5], Juan B. Salom [2,6], Jaime Masjuan [7,8], José Marco-Contelles [3] and Alberto Alcázar [1,*]

[1] Department of Research, Hospital U. Ramón y Cajal, IRYCIS, Ctra. Colmenar km 9.1, Madrid 28034, Spain; emma.martinez@hrc.es (E.M.-A.); alejandro.escobar@hrc.es (A.E.-P.); alberto.alcazar@hrc.es (A.A.)

[2] Unidad Mixta de Investigación Cerebrovascular, Instituto de Investigación Sanitaria La Fe - Universidad de Valencia, Valencia 46016, Spain; alicia_aliena@iislafe.es (A.A.-V.); torregrosa_ger@gva.es (G.T.); salom_jba@gva.es (J.B.S.)

[3] Laboratory of Medicinal Chemistry (IQOG, CSIC), C/Juan de la Cierva 3, Madrid 28006, Spain; m.chioua@csic.es (M.C.); jlmarco@iqog.csic.es (J.M.-C.)

[4] Center for Biomedical Technology, Universidad Politécnica de Madrid, Pozuelo de Alarcón 28223, Spain; rocio.fernandez@ctb.upm.es (R.F.-S.); daniel.gonzalez@ctb.upm.es (D.G.-N.)

[5] CIBER of Bioengineering Biomaterials and Nanomedicine (CIBER-BBN), Spain

[6] Departamento de Fisiología, Universidad de Valencia, Valencia 46016, Spain

[7] Department of Neurology, Hospital Universitario Ramón y Cajal, IRYCIS, Ctra. Colmenar km 9.1, Madrid 28034, Spain; jaime.masjuan@salud.madrid.org (J.M.)

[8] Department of Medicine, Facultad de Medicina, Universidad de Alcalá, Ctra. Madrid-Barcelona km 33.6, Alcalá de Henares 28871, Spain

Contents

A. Toxicokinetics study. Clinical biochemistry parameters (Table S1)……………………………S2

A. Toxicokinetics study. Clinical biochemistry parameters

Table S1

Clinical biochemistry parameters.

Females. Main allocation. Day 5. Mean ± SD

Day: 5 Relative to Start Date

Sex: Female		GLUC (mmol/L)	URE (mmol/L)	CRE (µmol/L)	CHOL (mmol/L)	TRIGL (mmol/L)	TP (g/L)	ALB (g/L)	GLOB (g/L)	BIL (µmol/L)	ALP (U/L)	ALT (U/L)	AST (IU/L)	GGT (U/L)
Group: A	Mean	7.84	7.12	27.82	3.05	0.78	55.04	35.67	19.37	0.52	93.24 a[1]	44.07	70.71 a[1]	0.00
	SD	0.57	0.82	1.39	0.37	0.12	0.65	2.18	2.04	0.23	16.49	6.84	4.52	0.00
	N	5	5	5	5	5	5	5	5	5	5	5	5	5
Group: B	Mean	7.08	6.90	28.64	3.18	0.86	55.80	35.39	20.41	0.81	86.67	54.64	105.37 d[2]	0.00
	SD	0.40	0.89	2.79	0.29	0.22	1.14	2.03	2.09	0.32	5.53	10.29	23.74	0.00
	N	5	5	5	5	5	5	5	5	5	5	5	5	5
Group: C	Mean	7.22	6.20	27.57	2.83	0.69	55.66	36.08	19.58	0.94	73.38 d[2]	50.43	92.48	0.00
	SD	0.72	0.83	1.23	0.32	0.26	2.97	2.59	2.22	0.19	8.03	6.39	13.60	0.00
	N	5	5	5	5	5	5	5	5	5	5	5	5	5
Group: D	Mean	6.77	7.08	27.58	2.90	0.72	55.22	34.32	20.89	0.96	80.71	57.27	116.10dd[3]	0.00
	SD	1.07	0.72	1.85	0.15	0.16	1.25	2.75	2.45	0.35	8.56	6.63	28.68	0.00
	N	5	5	5	5	5	5	5	5	5	5	5	5	5

1 [a - Group Factor Test: Analysis of Variance p < 0.05] 2 [d - Test: Dunnett 2 Sided p < 0.05]
3 [dd - Test: Dunnett 2 Sided p < 0.01]

Group A, Vehicle. Group B: QN23, 2 mg/kg/day. Group C: QN23 6 mg/kg/day. Group D: QN23 18 mg/kg/day
